# Supplementary material for: Tuning self-renewal in the Arabidopsis stomatal lineage by hormone and nutrient regulation of asymmetric cell division
Source: eLife. 2021 Mar 19;10:e63335. doi: 10.7554/eLife.63335 (PMC8009662; doi:10.7554/eLife.63335)
Supplement: Transparent reporting form [file elife-63335-transrepform.docx]

***eLife’s* transparent reporting form**

We encourage authors to provide detailed information *within their submission* to facilitate the interpretation and replication of experiments. Authors can upload supporting documentation to indicate the use of appropriate reporting guidelines for health-related research (see [EQUATOR Network](http://www.equator-network.org/%20)), life science research (see the [BioSharing Information Resource](https://biosharing.org/)), or the [ARRIVE guidelines](http://www.plosbiology.org/article/info:doi/10.1371/journal.pbio.1000412) for reporting work involving animal research. Where applicable, authors should refer to any relevant reporting standards documents in this form.

If you have any questions, please consult our Journal Policies and/or contact us: [editorial@elifesciences.org](mailto:editorial@elifesciences.org).

**Sample-size estimation**

- You should state whether an appropriate sample size was computed when the study was being designed
- You should state the statistical method of sample size computation and any required assumptions
- If no explicit power analysis was used, you should describe how you decided what sample (replicate) size (number) to use

Please outline where this information can be found within the submission (e.g., sections or figure legends), or explain why this information doesn’t apply to your submission:

- Information on sample size decision regarding POME measurement can be found in the “Image quantification” part of the “Material and Method” section.
- Sample size with lineage tracing experiments (n=2 leaves/condition; n>500 cells/condition) are limited by the labor-intensive manual cell labeling and tracking process.

**Replicates**

- You should report how often each experiment was performed
- You should include a definition of biological versus technical replication
- The data obtained should be provided and sufficient information should be provided to indicate the number of independent biological and/or technical replicates
- If you encountered any outliers, you should describe how these were handled
- Criteria for exclusion/inclusion of data should be clearly stated
- High-throughput sequence data should be uploaded before submission, with a private link for reviewers provided (these are available from both GEO and ArrayExpress)

Please outline where this information can be found within the submission (e.g., sections or figure legends), or explain why this information doesn’t apply to your submission:

- Information about replicates for each experiment is listed in each corresponding figure legend.
- Arabidopsis leaves do not have invariant lineages, nor can they be precisely synchronized. For lineage tracing experiments, leaves with similar starting cell number were chosen to minimize differences inherent in embryo development.
- For generating stomatal index counts, only high quality DIC images where cell outlines could be clearly seen were chosen and counted.

**Statistical reporting**

- Statistical analysis methods should be described and justified
- Raw data should be presented in figures whenever informative to do so (typically when N per group is less than 10)
- For each experiment, you should identify the statistical tests used, exact values of N, definitions of center, methods of multiple test correction, and dispersion and precision measures (e.g., mean, median, SD, SEM, confidence intervals; and, for the major substantive results, a measure of effect size (e.g., Pearson's r, Cohen's d)
- Report exact p-values wherever possible alongside the summary statistics and 95% confidence intervals. These should be reported for all key questions and not only when the p-value is less than 0.05.

Please outline where this information can be found within the submission (e.g., sections or figure legends), or explain why this information doesn’t apply to your submission:

- All the data points in boxplots and violin plots in all the figures are shown in each figure.
- Statistical testing methods are described in the figure legends of each figure where statistical tests were conducted, and described in detail in the “Statistical analysis” part of the “Material and Method” section.
- Unpaired Mann-Whitney tests are the default statistical testing method, to avoid assuming the normal distribution of the samples, except in experiments with small sample sizes (due to the high theoretical minimal of p-value in small sample sizes comparisons). Student’s *t*-tests are used in experiments with small sample sizes.
- All exact p-value are reported in each pairwise comparison in all the figures.

(For large datasets, or papers with a very large number of statistical tests, you may upload a single table file with tests, Ns, etc., with reference to sections in the manuscript.)

**Group allocation**

- Indicate how samples were allocated into experimental groups (in the case of clinical studies, please specify allocation to treatment method); if randomization was used, please also state if restricted randomization was applied
- Indicate if masking was used during group allocation, data collection and/or data analysis

Please outline where this information can be found within the submission (e.g., sections or figure legends), or explain why this information doesn’t apply to your submission:

- For qRT-PCR experiments, the group allocation information can be found in the “RNA extraction and qRT-PCR analysis” part of the “Material and Method” section.

**Additional data files (“source data”)**

- We encourage you to upload relevant additional data files, such as numerical data that are represented as a graph in a figure, or as a summary table
- Where provided, these should be in the most useful format, and they can be uploaded as “Source data” files linked to a main figure or table
- Include model definition files including the full list of parameters used
- Include code used for data analysis (e.g., R, MatLab)
- Avoid stating that data files are “available upon request”

Please indicate the figures or tables for which source data files have been provided:

- For POME analysis, the FIJI plugin, detailed user guide, and source code can be found in our method paper now published in New Phytologist (Gong et al., 2020). A brief description of this information is included in the “Image quantification” part of the “Material and Method” section.
